# Integration of Leprosy Elimination into Primary Health Care in Orissa, India

**DOI:** 10.1371/journal.pone.0008351

**Published:** 2009-12-18

**Authors:** M. Ruby Siddiqui, Nageswara Rao Velidi, Surendra Pati, Nilambar Rath, Akshay K. Kanungo, Amiya K. Bhanjadeo, Bandaru Bhaskar Rao, Bijaya M. Ojha, Kodyur Krishna Moorthy, Douglas Soutar, John D. H. Porter, Pemmaraju V. Ranganadha Rao

**Affiliations:** 1 London School of Hygiene and Tropical Medicine, London, United Kingdom; 2 LEPRA Health in Action, Bargarh, Orissa, India; 3 LEPRA Health in Action, Bhubaneswar, Orissa, India; 4 LEPRA Health in Action, Cherlapally, Hyderabad, India; 5 LEPRA Health in Action, Colchester, Essex, United Kingdom; 6 LEPRA Health in Action, West Marredpally, Secunderabad, India; Charité-Universitätsmedizin Berlin, Germany

## Abstract

**Background:**

Leprosy was eliminated as a public health problem (<1 case per 10,000) in India by December 2005. With this target in sight the need for a separate vertical programme was diminished. The second phase of the National Leprosy Eradication Programme was therefore initiated: decentralisation of the vertical programme, integration of leprosy services into the primary health care (PHC) system and development of a surveillance system to monitor programme performance.

**Methodology/Principal Findings:**

To study the process of integration a qualitative analysis of issues and perceptions of patients and providers, and a review of leprosy records and registers to evaluate programme performance was carried out in the state of Orissa, India. Program performance indicators such as a low mean defaulter rate of 3.83% and a low-misdiagnosis rate of 4.45% demonstrated no detrimental effect of integration on program success. PHC staff were generally found to be highly knowledgeable of diagnosis and management of leprosy cases due to frequent training and a support network of leprosy experts. However in urban hospitals district-level leprosy experts had assumed leprosy activities. The aim was to aid busy PHC staff but it also compromised their leprosy knowledge and management capacity. Inadequate monitoring of a policy of ‘new case validation,’ in which MDT was not initiated until primary diagnosis had been verified by a leprosy expert, may have led to approximately 26% of suspect cases awaiting confirmation of diagnosis 1–8 months after their initial PHC visit.

**Conclusions/Significance:**

This study highlights the need for effective monitoring and evaluation of the integration process. Inadequate monitoring could lead to a reduction in early diagnosis, a delay in initiation of MDT and an increase in disability rates. This in turn could reverse some of the programme's achievements. These findings may help Andhra Pradesh and other states in India to improve their integration process and may also have implications for other disease elimination programmes such as polio and guinea worm (dracunculiasis) as they move closer to their elimination goals.

## Introduction

The National Leprosy Control Programme was launched in India in 1955, using surveys, education and dapsone monotherapy to detect and treat leprosy cases. The programme was re-launched as the National Leprosy Eradication Programme (NLEP) in 1983 with the goal of elimination of leprosy as a public health problem (<1 case per 10,000). Multi-drug therapy (MDT), including rifampicin, clofazimine and dapsone for multibacillary (MB) leprosy patients and rifampicin and dapsone for paucibacillary (PB) leprosy, replaced dapsone monotherapy and the first phase of this vertical programme focussed on detecting and treating all leprosy cases. This successfully reduced the national prevalence of leprosy from 57.6 per 10,000 in March 1981 to 2.44 per 10,000 in March 2004 [1]. Leprosy was eliminated nationally by December 2005 [1]. With this target in sight the need for a separate vertical programme was diminished. The Government of India (GOI) initiated the second phase of the NLEP programme: decentralisation of the vertical programme, integration of leprosy services into the general health system (GHS) and development of an adequate surveillance system to monitor programme performance.

State governments developed a strategy to integrate vertical leprosy programmes into PHC in two stages, firstly integration of the functional components of leprosy services followed by merging of the infrastructure of NLEP. This process was facilitated by District Technical Support Teams (DTSTs) consisting of non-government organisation (NGO) medical officers, supervisors and support staff [2] and supported by the International Federation of Anti-Leprosy Associations (ILEP).

Functional integration included training PHC staff to, diagnose and manage leprosy and its complications, maintain MDT stocks, record and report cases and carry out information, education and communication (IEC) activities. Structural integration included placing leprosy-trained paramedical workers (PMWs) from the vertical programme into PHC clinics and establishing a District Nucleus (DN) of 4–7 members including, an assistant district medical officer for public health, a district nucleus medical officer (DN MO), a non-medical supervisor and 2 paramedical workers (some DNs also contained a laboratory technician and physiotherapist), to monitor leprosy programme activities.

After integration it was the responsibility of the GHS to ensure that leprosy was detected as early as possible, that correct treatment was given, that correct steps were taken to prevent disability where sensory loss and nerve damage were present and that all health workers, people affected by leprosy and the public were informed about leprosy [3]. The state of Orissa was one of the first in India to complete integration of leprosy elimination into GHS using the two-stage policy of functional and structural integration.

In 2004 a study by the National Institute of Health and Family Welfare (NIHFW) suggested that approximately 40% of all leprosy cases in India were either misdiagnosed (not true leprosy) or re-registered (previously released from treatment (RFT)) cases [4]. In response the GOI introduced a national policy of new case confirmation (‘validation’) in January 2005 [5]. This stated that all newly detected leprosy cases should be validated by a medical officer (MO) experienced in leprosy such as the DN MO or the DTST MO in the presence of the PHC MO before initiating MDT treatment. The aim of this policy was to reduce misdiagnosis and re-registration and to increase the leprosy diagnostic skills of PHC MOs. It was expected to be gradually phased out once misdiagnosis and re-registration rates were reduced to an acceptable level (<10%).

In order to evaluate the process of integration of leprosy services into GHS, an operations research study was carried out in the state of Orissa.

## Materials and Methods

### Ethics Statement

The Orissa State Ethical Committee convened the meeting on 19th June 2006 at 4pm under the chairmanship of Prof. Dr. S. K. Giri and approved the following protocol “Status of Leprosy Integration into Primary Health Care (PHC) in Orissa and Andhra Pradesh, India”

London School of Hygiene and Tropical Medicine Ethics Committee

Approval Form application number: 4046, “Status of Leprosy Integration into Primary Health Care (PHC) in Orissa and Andhra Pradesh, India”

Approval of this study is granted by the committee

Written informed consent was obtained from all participants in the study

### Study Site

Bargarh district in western Orissa had the highest prevalence of leprosy in India (5.3 per 10,000 in October 2005). It was also one of the most advanced districts in Orissa in terms of integration of leprosy services into GHS. Both functional and structural integration were completed in September 2004. Bargarh district was therefore selected as the site for this study. Leprosy elimination indicators for Bargarh district for 1997–2005 are shown in Table 1.

**Table 1:** Leprosy elimination indicators for Bargarh District 1997–2005.

Leprosy Elimination Indicator	1997–1998	1998–1999	1999–2000	2000–2001	2001–2002	2002–2003	2003–2004	2004–2005
**Prevalence rate (PR)-per 10,000**	41.9	15	37.7	21.6	24.8	20.7	8.18	6.17
**New case detection rate (NCDR)- per 10,000**	75.2	21.4	49.3	33.4	47.8	27	14.07	14.94
**Deformity proportion-%**	4.2	2.3	1.8	2.3	2.9	3.35	2.89	2.34
**Multibacillary (MB) proportion-%**	35.2	35.2	34.8	56	36.9	38.16	48.32	41.58
**Child proportion-%**	0	0	0	15	11.92	10.95	9.29	12.84

**Figure 1:**
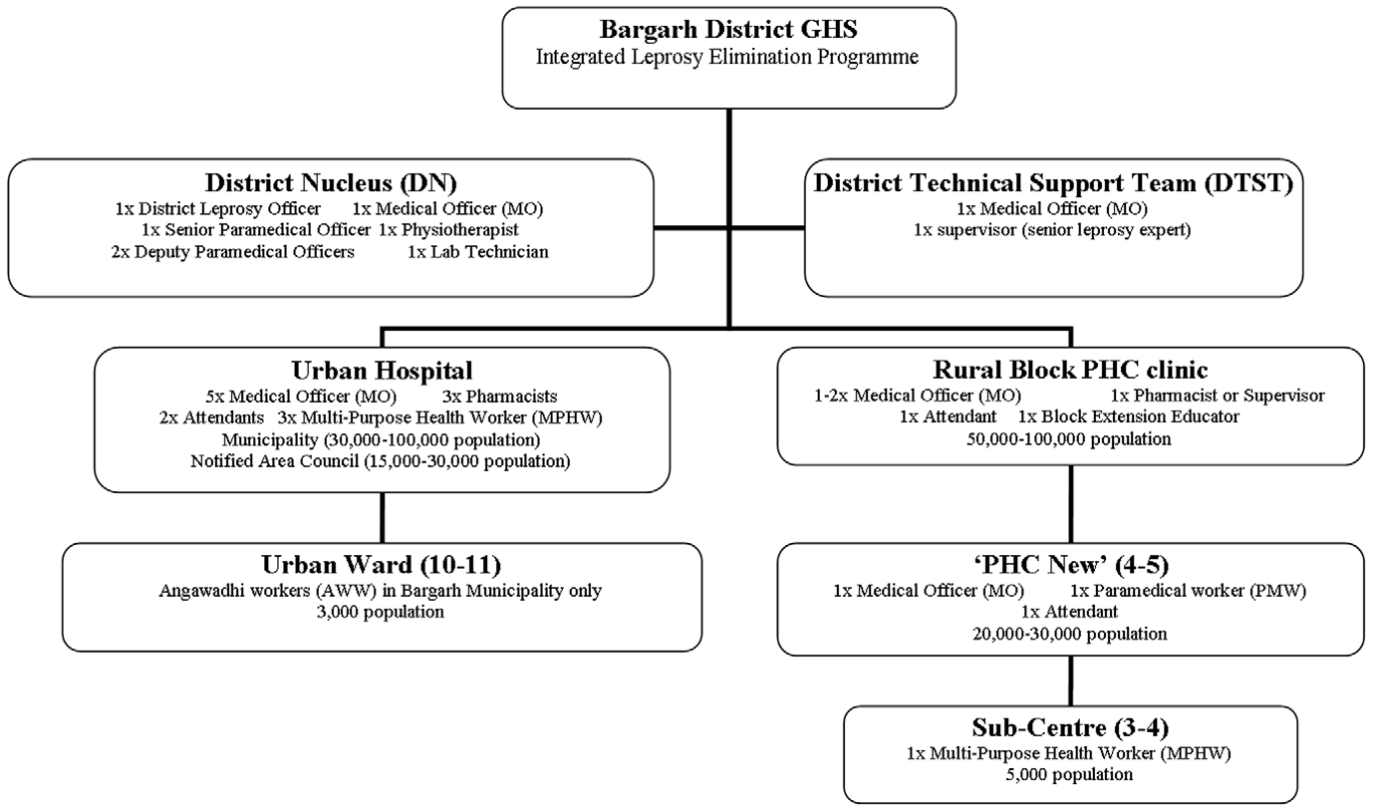
Infrastructure of the Bargarh District General Health System (GHS) and the Leprosy Elimination Programme.

There were 3 urban areas and 12 rural blocks in Bargarh district. An urban hospital or a rural ‘block’ PHC clinic provided services to a population of approximately 50,000–100,000 (Figure 1). A rural block was divided into 4–5 sectors, each containing a ‘PHC new’ clinic that covered 20,000–30,000 population. PHC clinics were managed by 1 or 2 medical officers (MO) with the help of a pharmacist or supervisor. Other staff at block or ‘PHC new’ level included a block extension educator responsible for information, education and communication (IEC) activities and health programme data, attendants that also dressed wounds and male or female multi purpose health workers (MPHW). Each sector contained 3–4 Sub-Centres which were satellite PHC clinics staffed by a MPHW. Each Sub-Centre covered a population of 5,000.

Urban areas were termed municipalities or notified area councils (NACs) depending on the population size (30,000–100,000 and 15,000–30,000 respectively) and were further divided into approximately 11 urban wards. An urban ward was equivalent to a rural village in terms of population size (2,000–3,000). However the urban health infrastructure was less developed than the rural health structure so there was no further subdivision of urban hospital medical services, although Bargarh municipality expanded its Integrated Child Development Scheme that used pre-school teachers (Angawadhi workers or AWW) to implement health, nutrition and immunisation programmes for children and pregnant women to also include leprosy, tuberculosis and malaria control.

LEPRA Society is a leprosy-oriented NGO with several active leprosy elimination projects in the states of Orissa, Andhra Pradesh., Bihar and Madhya Pradesh. In Bargarh district LEPRA Society provided: prevention of disability (POD) and ulcer management services, protective microcellular rubber (MCR) footwear and adaptive devices, and organised referrals for reconstructive surgery. In response to the lack of disability and ulcer care services within the GHS, LEPRA Society initiated the Integrated Community Health Project (ICHP) in some areas of Bargarh, such as Attabira rural block, Bheden rural block and Bargarh municipality. The project provided demonstrative training camps for patients, families and communities through community health promoters and disability care clinics which were designed to help the patient become self-reliant.

A total of 8 areas in Bargarh district: 3 urban areas (Bargarh, Barpali and Padampur), and 5 rural blocks (Attabira, Agalpur, Bijepur, Jamala and Katapali) were selected for this study. In addition Sambalpur municipality in neighbouring Sambalpur district was included in the study (giving a total of 4 urban areas). This was to ensure a minimum of 5 urban PMWs were included in the study due to their unique perspective of the leprosy programme before and after integration (see Section 2.2).

### Subjects

A total of 173 subjects were recruited from urban areas and rural blocks, including 30 leprosy patients, 64 health care providers, 10 PMWs and 69 members of the local communities (Table 2).

**Table 2:** Subjects included in the leprosy integration operational research study.

	Urban					Rural						
	Bargarh ICHP	Barpali	Padampur	Sambalpur	Total	Attabira ICHP	Agalpur	Bijepur	Jamla	Katapali	Total	Grand Total
**Interviews:**												
**Patients:**	**3**	**1**	**6**	**3**	**13**	**6**	**2**	**5**	**2**	**2**	**17**	**30**
Undergoing MDT	1		2	2	5	1	1	2	1		5	10
Defaulter			1		1						0	1
RFT	2	1	3	1	7	5	1	3	1	2	12	19
**Providers**	**8**	**3**	**6**	**4**	**21**	**6**	**6**	**6**	**4**	**4**	**26**	**47**
MO	3	1	4	2	10	3	2	2	1	1	9	19
Pharmacist	2	1	1	1	5	1	2	1	1	1	6	11
ANM					0	1	1	2	1	1	6	6
AWW	1				1						0	1
PMW	2	1	1	1	5	1	1	1	1	1	5	10
**Focus groups:**												
PHC staff	**3**	**0**	**3**	**0**	**6**	**6**	**5**	**5**	**2**	**3**	**21**	**27**
Supervisor or LHV	1		1		2	2	3	1	1	1	8	10
MPHW or ANM	1		1		2	3	1	3		1	8	10
BEE					0	1	1	1		1	4	4
Attendant (dresser)	1		1		2				1		1	3
**Community:**	**10**	**4**	**0**	**5**	**19**	**0**	**5**	**40**	**5**	**0**	**50**	**69**
**Total Subjects:**	**24**	**8**	**15**	**12**	**59**	**18**	**18**	**56**	**13**	**9**	**114**	**173**

The sample size was dictated by the data collected. Recruitment continued until ‘theoretical saturation’ occurred (no new themes emerged in the data analysis) [6]. At least 5 members of each subject group were included for both urban and rural areas.

### Data Collection

Firstly leprosy records and registers were reviewed to evaluate programme performance in the selected PHC clinics. Secondly, using a qualitative approach, the issues raised by the integration process and the perceptions of patients and providers in relation to leprosy services were assessed. Patients and communities were asked to comment on the resources available to them during and after MDT and on any constraints in accessing PHC. Providers were asked to describe issues raised by the introduction of an additional health programme into PHC and the level of guidance and support received. In addition we spoke to PMWs about their new role in GHS and within the leprosy elimination programme.

### Record Review

#### Simplified Information System (SIS)

The Simplified Information System (SIS) was a system of record and report formats that facilitated data analysis at each PHC level. It consisted of 4 leprosy format (LF) forms: the patient case card (LF1), patient treatment record (LF2), MDT stock card (LF3) and monthly reporting form (LF4). One LF1 per patient containing patient details, diagnosis results and monthly treatment dates was maintained by the MPHW at the Sub-Centre level. LF2 and LF3 were records of all leprosy patients plus treatment dates and MDT stock levels within a block or urban area. These forms were contained within the master register which was maintained at block or hospital level. A similar register was maintained at the sector level. LF4 was used to report monthly leprosy indicators for each block or urban area to the District Nucleus. High leprosy indicators suggested areas that may require additional elimination activities (Table 1). The prevalence rate was used to monitor the progress of the local elimination programme, the new case detection rate, MB proportion and child case proportion may indicate high transmission areas, and disability proportions higher than zero may indicate a delay in diagnosis.

We reviewed leprosy records and registers at each PHC clinic for the period 1^st^ Dec 2004-1^st^ Dec 2005, to evaluate programme performance. In addition to leprosy indicators, the number of leprosy cases that were misdiagnosed, re-registered or defaulted from MDT was also deduced.

#### Suspect referral forms (new case validation)

New case validation was implemented in Orissa on 1^st^ April 2005. The PHC MO would make the initial diagnosis and then refer the suspect case for validation using a suspect referral form. Part 1 of the form remained at the PHC clinic and Part 2 was sent to the validating MO. The suspect referral form did not form part of the SIS system. The suspected case was usually seen by the validating MO within 1 month. If leprosy was confirmed, the patient was given the first dose of MDT and either registered and referred to the appropriate Sub-Centre for continuation of treatment or sent to his/her local PHC clinic.

We reviewed the suspect referral book and cross-matched with the master register. The proportion of suspect cases; misdiagnosed, put under observation (indeterminate diagnosis) or re-registered was deduced. The proportion for which validation was pending more than one month after referral was also deduced.

### Non-Participant Observation

Non-participant observation [7] was used to observe general PHC activities and to observe the management of leprosy cases (if present). Four major areas were examined: infrastructure of the PHC clinic, the work of the PHC staff (especially in relation to leprosy patients), the flow of patients through the clinic and the performance of programme requirements (such as completion of registers, patient care and supervision of treatment).

### Semi-Structured Interviews

Semi-structured open-ended interviews [7] were carried out with leprosy patients, health care providers and PMWs. Members of DTSTs were also interviewed but have been removed from the study as insufficient numbers were interviewed to ensure theoretical saturation and anonymity.

### Focus Group Discussions

Focus group discussions [7] were conducted in the communities to supplement observations in leprosy patient interviews concerning local health-seeking behaviour and beliefs. Focus group discussions were also conducted with PHC staff such as: MPHWs, supervisors, Block extension educators and attendants (dressers) in order to broaden the range of health care providers included in the study and supplement some of the findings from provider interviews.

### Analysis Methods

The qualitative data was analysed using thematic analysis [6]. This involved identifying common themes and examples of these themes until ‘theoretical saturation’ was achieved (no new themes emerged from the data). Validity was established by method triangulation (similar conclusions in non-participant observations, semi-structured interviews and focus groups) [8].

## Results

### Quality of Leprosy Services

#### Diagnosis and treatment

The quality of leprosy diagnosis and treatment services was generally of a high standard. All PHC staff were knowledgeable about the signs and symptoms of leprosy and methods of diagnosis. MDT treatment procedures and recording on patient cards were also followed correctly. Almost all PHC staff expressed satisfaction with the quality and frequency of training provided during functional integration.

MDT adherence was high in Bargarh district (the average defaulter rate was 3.83%), illustrating the high quality of leprosy treatment and counselling services in GHS (Table 3, Table 4).

**Table 3:** Leprosy, MDT adherence and Validation indicators in Bargarh district, Orissa (Rural).

	Rural											
	Attabira-ICHP		Agalpur		Bijepur		Jamala		Katapali		Mean Rural	
	Number	Rate or %	Number	Rate or %	Number	Rate or %	Number	Rate or %	Number	Rate or %	Mean	Rate or %
**Leprosy indicators**												
PR (per 10,000)	247	15.23	237	22.66	159	15.21	155	13.35	152	9.59	**190.00**	**15.21**
NCDR (per 10,000)	106	6.54	120	11.47	62	5.93	75	6.46	133	8.40	**99.20**	**7.76**
MB proportion (%)	147	59.51%	124	52.32%	106	66.67%	94	60.65%	68	44.74%	**107.80**	**56.78%**
Child proportion (%)												
**MDT adherence indicators**												
Proportion RFT by NLEP guidelines	154	97.47%	140	87.50%	119	100.00%	91	95.79%	50	90.91%	**110.80**	**94.33%**
Defaulter rate	4	1.62%	20	8.44%	0	0.00%	4	2.58%	5	3.29%	**6.60**	**3.19%**
**Validation indicators**												
Misdiagnosis	1	2.78%	0	0.00%	0	0.00%	0	0.00%	1	2.38%	**0.40**	**1.03%**
Validation pending	2	5.56%	14	66.67%	3	27.27%	7	28.00%	13	30.95%	**7.80**	**31.69%**
Under observation	1	2.78%	0	0.00%	1	9.09%	2	8.00%	1	2.38%	**1.00**	**4.45%**
Re-registration	1	2.78%	2	9.52%	0	0.00%	2	8.00%	7	16.67%	**2.40**	**7.39%**

Data was from 1st Dec 2004-1st Dec 2005. N.B. Leprosy indicators represent means for the period under study not the final value.

**Table 4:** Leprosy, MDT adherence and Validation indicators in Bargarh district, Orissa (Urban).

	Urban										Grand total	
	Bargarh-ICHP		Barpali		Padampur		Sambalpur		Mean Urban		Mean Urban & Rural	
	Number	Rate or %	Number	Rate or %	Number	Rate or %	Number	Rate or %	Mean	Rate or %	Mean	Rate or %
**Leprosy indicators**												
PR (per 10,000)	126	21.47	48	24.30	34	20.51	63		**67.75**	**22.09**	**135.67**	**17.79**
NCDR (per 10,000)	58	9.89	34	17.21	26	15.68	62		**45.00**	**14.26**	**75.11**	**10.20**
MB proportion (%)	70	55.56%	30	62.50%	23	67.65%	40	63.49%	**40.75**	**62.30%**	**78.00**	**59.23%**
Child proportion (%)												
**MDT adherence indicators**												
Proportion RFT by NLEP guidelines	75	84.27%	22	100.00%	12	85.71%	24	96.00%	**33.25**	**91.50%**	**76.33**	**93.07%**
Defaulter rate	14	11.11%	0	0.00%	2	5.88%	1	1.59%	**4.25**	**4.65%**	**5.56**	**3.83%**
**Validation indicators**									**Excluding Sambalpur**		**Excluding Sambalpur**	
Misdiagnosis	4	6.67%	10	23.81%	0	0.00%	3	5.36%	**4.67**	**10.16%**	**2.00**	**4.45%**
Validation pending	25	41.67%	3	7.14%	1	4.55%	2	3.57%	**9.67**	**17.78%**	**8.50**	**26.48%**
Under observation	1	1.67%	1	2.38%	2	9.09%	14	25.00%	**1.33**	**4.38%**	**1.13**	**4.42%**
Re-registration	0	0.00%	1	2.38%	0	0.00%	4	7.14%	**0.33**	**0.79%**	**1.63**	**4.92%**

Data was from 1st Dec 2004-1st Dec 2005 except Sambalpur (data from 1st April 2005-1st Dec 2005). N.B. Leprosy indicators represent means for the period under study not the final value.

#### Registration of leprosy cases and MDT stock management

Patient cards, sector registers and master registers were well maintained in all PHC clinics. However there was much variability in responsibility for the master treatment register at block or hospital level. Usually the pharmacist or PMW managed the register but occasionally supervisors, Block extension educators or the DN non-medical supervisor took responsibility. This was particularly evident in urban hospitals where medical staff were extremely busy and the PMW or district nucleus therefore assumed many of the leprosy programme responsibilities. Although this did not appear to affect the quality of SIS, urban PHC staff acknowledged a minimal understanding of leprosy management indicating capacity-building of urban PHC staff was compromised. Conversely block or hospital MDT stock management was almost always managed by, and managed effectively, by the pharmacist.

The timing of defaulter tracing varied from 2–3 days to 2 months and was generally performed quicker in rural areas. The following illustrates the importance of defaulter tracing, continuous counselling and patient follow-up:

*Patient 3E, 35 years old, was diagnosed with MB leprosy 4 years ago after developing clawing of his right hand and anaesthesia in his right foot. The MO explained about leprosy, the importance of regular treatment and how to carry out self-care. He was a poor man and had to find work so he stopped taking MDT after 6 months and travelled back to his home village (he did not know that he could take the remaining treatment with him). He did not inform the hospital and does not think they tried to trace him. He has since developed painful neuritis in his hand and a maggot-infested tropic ulcer on his right toe. He was charged 200 rupees for antibiotics which he could not afford and the toe was amputated. He was back now but too scared to restart MDT in case he was given more bad news.*

#### Validation

The basis of our evaluation of the validation system was the suspect referral book at block or urban hospital level. The proportion of suspect cases successfully validated, misdiagnosed, under observation, re-registered or with validation still pending was deduced from this book. However the suspect referral book was not included in the SIS system. Therefore the PHC clinics had no clear guidelines for recording the validation process. The result of validation was generally noted on Part 2 of the suspect referral form which the validating MO signed and returned to the PHC pharmacist or PMW. If the suspect case was not seen, the validating MO retained Part 2. If the validating MO, PHC pharmacist or PHC PMW did not note the result of validation on Part 1 of the form, still attached to the suspect referral book, there would be no validation record at the PHC clinic as Part 2 was often given to the patient as a record of the result (and to be used as a personal treatment card). If the suspect case did not appear in the master register as a confirmed leprosy case it was not clear whether the patient had been seen for validation or whether validation was still pending.

Generally the validation result was noted on Part 1 or it was possible to deduce using the master register. A missing result was noted as ‘validation pending’ for this study although it was possible that some cases had been validated but had not registered and initiated treatment. Sambalpur was removed from the validation analysis (although the numbers observed are shown in Table 4) as completion of suspect referral forms was carried out on the validation dates (i.e. for those suspect cases that had attended the validation session) instead of at the time of diagnosis. Therefore Sambalpur had no record of the true number of suspect cases for which validation was pending.

Mean misdiagnosis and re-registration were 4.45% (95% CI 0.46%–8.45%) and 4.92% (95% CI 3.23%–9.89%) respectively (Table 3, Table 4), lower than that suggested by the NIHFW [4], although particular attention should be paid to Barpali (mean misdiagnosis = 23.81%), Katapali (mean re-registration = 16.67%) and Sambalpur (mean proportion of suspect cases under observation = 25.0%). Misdiagnosis appeared to be higher in urban areas, 10.16% (95% CI 3.72%–16.59%), than rural blocks, 1.03% (95% CI 0%–3%), P = 0.001 although the opposite appeared to be the case for re-registration, rural, 7.39% (95% CI 3.74%–11.05%) versus urban, 2.38% (single observation), P = 0.008. Urban areas may thus benefit from increased training in leprosy diagnosis while increased counselling at RFT may be required in rural blocks.

The most striking observation was the proportion of suspect cases for which validation was still pending more than 1 month after initial diagnosis, 26.48% (95% CI 21.42%–31.53%). This was particularly evident in rural blocks, 31.69% (95% CI 24.78%–38.60%) compared with urban areas, 17.78% (95% CI 10.24%–25.33%), P = 0.01 and some cases dated back to April 2005 (although the majority, 21.4%, were awaiting validation 1–3 months after initial diagnosis). It was possible that recording error was responsible for some of these observations or that suspect cases were validated elsewhere. However follow-up of these cases was critical.

Validation has two objectives, firstly to reduce the proportion of misdiagnosis and re-registration and secondly to improve the leprosy diagnosis skills of PHC MOs by performing validation in their presence. However PHC MOs, particularly in urban areas, were often not present at validation and admitted that they had little further knowledge of patients after referring for validation. In addition, in an effort to reduce the multiple visits required of patients, PMWs and MPHWs often referred suspect cases directly to validation, bypassing the PHC MO. This reduced exposure of the PHC MOs to leprosy patients and reduced their capacity to diagnose leprosy. The following illustrates how the validation process can delay initiation of treatment and could lead to non-validation.

*Patient 6C, 25 years old, was diagnosed with MB leprosy 6 months ago after noticing anaesthetic patches on his chest, back and knee and joint pains. He knew from TV and posters that this could be leprosy so he went straight to his local ‘PHC new’ clinic. However he was referred to the block PHC 8 km away for validation. The validating MO did not come so he was sent to another PHC clinic 12 km away for validation. Again he missed the validating team and was sent to the medical college 20 km away for validation. He was told that they were not permitted to validate his diagnosis. His diagnosis was eventually confirmed nearly 2 months after the initial diagnosis.*

#### Counselling

Counselling of leprosy patients should be carried out at all stages of the programme including at diagnosis, during treatment and at RFT. This ensures MDT adherence, allays fears and warns of possible drug side effects or complications. At RFT it was important to reassure patients if anaesthesia or deformities have not disappeared as these patients may otherwise try to obtain more MDT from another PHC clinic. Re-registration of RFT cases incorrectly raises the leprosy prevalence rate.

All PHC staff were knowledgeable in counselling messages. However initial counselling was generally carried out by validating staff, particularly in urban hospitals. In rural areas counselling during MDT and at RFT was usually carried out at the Sub-Centre level by MPHWs. However in urban hospitals or busy block PHCs, pharmacists often had no time to counsel patients. There appeared to be no further follow-up of patients post-RFT unless LEPRA Society was active in the area.

*Patient 3F, 42 years old was diagnosed with PB leprosy 1 year ago. He came to the urban hospital monthly for MDT but saw little improvement in the anaesthetic patch. He discontinued MDT for 2 months but then decided to continue. He received the remainder but saw no further improvement. He was not told that he had completed the course until a month later when he returned for his next dose. He wanted to continue because the patch was still anaesthetic but was told he couldn't. He believes treatment should continue until symptoms disappear. Now he feels it may get worse. He has since developed a silent ulcer on his foot. He thinks that he was diagnosed incorrectly and it wasn't leprosy because the treatment didn't work.*

It was important that patients were advised to check families for symptoms as there were no longer active searches or surveys. New case detection now relied on voluntary reporting. In the rural system MPHWs often examined families in the home during patient follow-up visits. Otherwise patients were advised to refer family members with leprosy symptoms for examination. In the urban system patients were not usually advised about family members (except by AWWs in Bargarh municipality).

#### Management of complications

PHC staff recognised the signs and symptoms of leprosy reactions such as Type I and II reactions (reversal reaction and erythema nodosum leprosum respectively) and neuritis and immediately referred patients to PHC MOs for prednisolone treatment. Reaction cases were generally followed up by MPHWs in rural blocks and PMWs in urban areas.

Prevention of disability (POD) and ulcer care however were much more variable. MOs would either advise self-care, prescribe antibiotics or refer patients to the attendant for dressing. MPHWs generally advised self-care but few carried out ulcer care. This may be due to lack of materials or lack of training. LEPRA Society and more recently GOI have organised POD camps in Bargarh district. These camps demonstrated practical skills in ulcer care and POD methods. The following illustrates how effective self-care and exercises can be in preventing disability:

*Patient 7D, 29 years old, was diagnosed with MB leprosy after noticing some weakness in her right little finger. She started MDT and was advised to soak her hands and apply oil daily. She was given exercises to carry out daily, morning and night, to straighten the hand. She has been determined to prevent this disability and has followed the advice consistently. After 6 months she no longer has a claw hand but some weakness remains.*

There may be an issue of stigma in relation to ulcer and POD care. Some MPHWs in rural blocks preferred not to treat ulcers (although this may not be related to leprosy stigma). There were claims that in urban hospitals the MOs or other PHC staff tried to dismiss leprosy patients as quickly as possible to avoid unease amongst other patients, medical staff (including dressers) and the leprosy patients themselves.

*Provider 1GH, 34 years old, said that MOs *(in an urban hospital) *were extremely busy and usually had no time to explain leprosy to patients and convince them to take MDT. Also there was still stigma. Other patients would not stand next to leprosy patients. The MO immediately referred leprosy patients to the district nucleus. Ulcer cases did not generally come to the hospital. They usually went to nearby mission hospitals because there were no dressing facilities or the dressers would not dress leprosy patients, probably because of stigma because they would dress other wounds. Maybe other patients complain and tell him not to do it *(this was denied by the dresser).

In urban areas, patients with deformities or disabilities could be referred to the orthopaedic surgeon within the hospital. However in rural areas where LEPRA Society was not active there was little knowledge of treatment options for patients with deformities. Socioeconomic rehabilitation advice was a much neglected area of leprosy care but often the most important to the patient. LEPRA Society and some PHC blocks were attempting to rectify this by helping patients apply for GOI pensions for the handicapped (a 3% quota has been allocated to leprosy). RFT certificates were issued by the PHC clinics to help with the application process.

*Patient 3C, 34 years old, was diagnosed with MB leprosy 3 months ago after noticing anaesthesia and abnormality of her right foot. She has been unable to work since developing an ulcer there. She was very poor and has a very small house. She needs any assistance to help buy rice. There was a ‘Below Poverty Line’ (BPL) card that would be helpful but she doesn't have one. The Panchayat (local administrative council) did not record her in the book. Her eldest son has developed a hernia and was in pain and needs surgery but she cannot afford it. Nobody is listening.*

### Leprosy Awareness

It was the responsibility of GHS to ensure that all health workers, people affected by leprosy and the public are informed about leprosy. This was achieved through leprosy training, counselling and IEC campaigns.

#### IEC (community awareness)

Current GOI strategy was to carry out high profile IEC campaigns once or twice per year, using a range of methods such as TV, radio, posters, pamphlets, IEC vans, film shows and folk dances. These were effective in creating leprosy awareness in the community and encouraging new cases to come forward for detection. The low disability rate in Bargarh district (2.34% in October 2005) indicated that diagnosis was occurring at an early stage. In addition stigma appeared greatly reduced particularly in rural areas where patients were no longer ostracised from communities.

Between IEC campaigns there was very little IEC activity unless LEPRA Society was active in the area. MPHWs (and AWWs in Bargarh municipality) occasionally used flash cards in small meetings and carried out IPC (interpersonal communication) during field visits. However they had many responsibilities and often had too little time to carry out effective IEC.

#### Training (provider awareness)

Leprosy training during functional integration and subsequent capacity-building by DTSTs was generally considered thorough, useful and interesting. Most PHC staff felt they had gained from their increased knowledge and expertise in leprosy. The only area lacking and in which they wanted more practical experience was ulcer, POD and disability care. No evaluation, however, of training efficacy has been carried out.

## Discussion

There was overwhelming support and approval for integration of the leprosy elimination programme into GHS. Patients could now obtain treatment and medical care easier and there was less stigma now that patient care was incorporated into the GHS. PHC staff felt they were now more knowledgeable about leprosy and able to serve their communities better. The two-stage strategies of functional and structural integration had built a strong knowledge-base and capacity within the PHC and provided a support network of leprosy experts.

Even PMWs, who might be expected to be less supportive, generally felt the integrated programme was better for patients and for leprosy control. Previously it had been difficult for them to cover the whole population and perform effective patient follow-up. PMWs appeared to play a central role in the newly integrated programme. They facilitated leprosy diagnosis and SIS management as well as supervising patient follow-up, new case detection and IEC.

However the relinquishing of leprosy programme responsibilities by urban PHC MOs to the District Nucleus in Bargarh and Padampur hospitals, to the validating MO in Padampur hospital and to the PMW at Barpali urban community health centre (who had his own weekly leprosy clinic), indicated that a vertical structure within the integrated system had developed. Capacity-building of hospital staff had been compromised and PMWs, DNs and validating MOs had been diverted away from their own responsibilities to carry out leprosy case management. Urban PHC MOs admitted having little knowledge of their patients after suspect referral. They generally did not attend validations and did not follow up reaction patients. Some pharmacists in urban hospitals, particularly Sambalpur hospital, had minimal involvement in the leprosy programme.

Validation of suspect leprosy cases was introduced to reduce misdiagnosis and re-registration and intended to be a transitional scheme until MOs were deemed to be fully trained in leprosy diagnosis. However this policy had inadvertently created a larger problem in Bargarh district, a high proportion of validations pending (26.48%), between 1 and 8 months after initial diagnosis.

Leprosy patients waiting to start MDT 1–8 months after diagnosis could have serious implications to the leprosy elimination programme. It could reverse some of the programme's achievements by reducing early diagnosis and increasing disability rates. It could also be keeping the prevalence rate artificially low because validations still pending do not appear in any indicators. This may explain why some members of the health provider community suspected that the process of validation was introduced to ensure that the elimination target was met. But most importantly it would be harmful to the patient if, despite seeking appropriate health care within the GHS, he/she failed to be properly diagnosed.

In response to this study's findings the DTST launched an investigation and traced 55 of the 70 suspect cases for which validation was pending. The main reasons for non-validation appeared to be salary, stigma and distance. Many of these cases were casual workers and survived on daily salaries. Salary and distance appeared to be particularly important in rural blocks but salary and stigma were key issues in urban areas (data not shown).

The policy of validation needed immediate modification. It was clear that, despite introducing a specific suspect referral book (other states have no recording system for validation) stricter procedures for the recording of new case validation were required. Alternatively suspect cases could be allowed to start MDT before validation or responsibility for leprosy diagnosis could be restored to the PHC MO and monitored through validation of a sample of all new cases. Particular attention could then be paid to PHC clinics with high misdiagnosis or re-registration rates. In response to this study, the state of Orissa modified the validation policy to allow PHC MOs to carry out validations with strict monitoring by the local DN.

The emphasis of the leprosy elimination programme has been diagnosis and treatment in order to achieve the elimination target. Now that that target has been achieved in India (0.95 per 10,000 in December 2005), the GOI recognised the need to turn its attention to POD and ulcer care, rehabilitation and referral systems [9]. Increased training and practical experience in government-sponsored POD camps has already started. Demonstrations by PHC staff in effective ulcer and POD care, as performed by the LEPRA Society ICHP, will help patients and communities to become self-reliant.

A referral centre or referral system would be useful for all complications of leprosy such as severe reactions, ulcers and disabilities requiring surgery, physical rehabilitation and social and economical rehabilitation. Surgery referrals were mainly carried out by LEPRA Society in Bargarh district but physiotherapy and social and economical rehabilitation were rare. Such a referral centre could be developed in association with LEPRA Society and other NGOs to incorporate other health conditions that lead to deformities and disabilities such as diabetes, genetic disorders or physical injury.

The state of Orissa successfully implemented integration of an elimination programme into their primary health care system. Continuous monitoring of the programme and timely action enabled the programme to adapt quickly to the PHC environment. Similarly the programme quickly responded to issues arising from this study relating to new case validation and will monitor issues of POD and ulcer care and urban health care structures. These findings may help Andhra Pradesh and other states in India to improve their integration process and may also have implications for other disease elimination programmes such as polio and guinea worm (dracunculiasis) as they move closer to their elimination goals.
